# Comparative analysis of intestinal microbiota composition and transcriptome in diploid and triploid *Carassius auratus*

**DOI:** 10.1186/s12866-022-02709-5

**Published:** 2023-01-02

**Authors:** Yidan Cai, Ke Wei

**Affiliations:** Medical College, Hunan University of Chinese Medicine, Changsha, 410208 Hunan China

**Keywords:** Triploidization, Disease resistance, Intestinal microbiome, 16S rRNA sequencing, mRNA-sequencing

## Abstract

Polyploidy and the microbiome are crucial factors in how a host organism responds to disease. However, little is known about how triploidization and microbiome affect the immune response and disease resistance in the fish host. Therefore, this study aims to identify the relationship between intestinal microbiota composition, transcriptome changes, and disease resistance in triploid *Carassius auratus* (3nCC). In China’s central Dongting lake water system, diploid (2nCC) and triploid *Carassius auratus* were collected, then 16S rRNA and mRNA sequencing were used to examine the microbes and gene expression in the intestines. 16S rRNA sequencing demonstrated that triploidization altered intestinal richness, as well as the diversity of commensal bacteria in 3nCC. In addition, the abundance of the genus *Vibrio* in 3nCC was increased compared to 2nCC (*P* < 0.05). Furthermore, differential expression analysis of 3nCC revealed profound up-regulation of 293 transcripts, while 324 were down-regulated. Several differentially expressed transcripts were related to the immune response pathway in 3nCC, including *NLRP3*, *LY9*, *PNMA1*, *MR1*, *PELI1*, *NOTCH2*, *NFIL3,* and *NLRC4*. Taken together, triploidization can alter bacteria composition and abundance, which can in turn result in changes in expression of genes. This study offers an opportunity for deciphering the molecular mechanism underlying disease resistance after triploidization.

**Supplementary Information:**

The online version contains supplementary material available at 10.1186/s12866-022-02709-5.

## Background

Polyploidy has an important role in the disease resistance of fish [1]. For instance, in Atlantic salmon (*Salmo salar L.*), triploids may be at a disadvantage compared with their diploid siblings in their defense against bacterial infections [2]. Similarly, in response to a challenge with *Vibrio anguillarum*, the mortality of triploid Chinook salmon (*Oncorhynchus tshawytscha*) increased [3]. Dégremont et al. indicated that triploid oysters have higher disease resistance than diploids, as observed in *Crassostrea virginica* and *Saccostrea glomerata* [4]. In fertile triploid fish, normal gonadal development causes the energy needed for somatic growth to be channeled into gamete production. Consequently, this results in adverse effects, including poor physical growth and flesh quality, and can increase mortality and morbidity rates [5, 6]. Nevertheless, this phenomenon did not occur in the *Carassius auratus* complex in the water system of Dongting Lake, which is manifested as triploid *Carassius auratus* (3nCC) populations exhibited less sensitivity to environmental change than diploid *Carassius auratus* (2nCC) populations [7]. Consequently, it is crucial to understand how triploidy influences disease resistance in the *Carassius auratus* complex.

Intestinal microbial communities of animals are extremely diverse and active [8]. Microorganisms inhabiting the gastrointestinal tract, such as bacteria, archaea, viruses, fungi, and microeukaryotes, make up the intestinal microbiota [9, 10]. The intestinal microbiome is a source of key enzymes essential to food digestion and nutrient absorption for the host [11]. Technology development for next-generation sequencing (NGS) has enhanced the understanding of the diverse and complex communities of microbial symbionts residing within hosts. The diverse and dynamic microbial community of the gastrointestinal tract plays a critical role in modulating the host’s health and nutrition [12], immunity and defense against pathogens [13], growth and development [14], and behavior [15].

The intestinal microfloras are also closely associated with disease resistance in host animals and play a key role in the host’s immune system’s induction, education, and functionality [16, 17]. It has been demonstrated that *Se-rich B. subtilis* improves intestinal microbial changes induced by Hg, reduces *Aeromonas* abundance and inflammation in common carp [18]. Liu et al. found that Pb accumulated in the gut causes dysbiosis of the microbiota, affects intestinal immunity and digestion, and damages the silver carp’s intestinal barrier [19]. Shi et al. suggested antibiotics administered to grass carp would exacerbate oxidative stress, lead dysbiosis of intestinal bacteria, inhibit the immune system of the mucosa, and activate apoptosis [20]. According to Qiao et al., poly-hydroxybutyrate may have beneficial effects on immunity and disease resistance through its interaction with the gut microbiota [21]. In tilapia, NE can affect immunity indirectly by means of microbial changes as well as directly by stimulating host tissue [22]. There are potential dietary implications to altering the intestinal microbiota because the microbiota aid digestion and conversion of complex plant molecules into short-chain fatty acids are necessary for daily energy metabolism [23]. To develop and function properly in zebrafish, the immune system depends on bacteria and their products [24]. However, the role of the intestinal microbiome in the disease resistance of the triploid *Carassius auratus* has not been fully elucidated.

Considering the information available from previous research, we hypothesized that triploidization might change the intestine bacterial community, boost immunity, and increase disease resistance in the triploid *Carassius auratus*. To test this hypothesis, the composition of intestinal microbiota between diploid and triploid *Carassius auratus* was conducted. Additionally, mRNA-seq revealed many transcripts that differed between diploid and triploid *Carassius auratus*.

## Results

### Differences of richness and diversity of the microbiota between diploid and triploid of *Carassius auratus*

There were 453,811 good-quality 16S rRNA gene sequences obtained. A notable number of 400,890 sequences (88.34%) was associated with 1556 OTUs. OTUs were categorized into 34 phyla, 79 classes, 182 orders, 312 families, 594 genera, and 646 species based on the number of effective OTUs (Table 1).

**Table 1:** Species annotation statistics

Sample	Kindom	Phylum	Class	Order	Family	Genus	Species
2nCC_1	1	21	47	120	209	389	420
2nCC_2	1	27	57	132	216	408	437
2nCC_3	1	22	51	122	209	380	411
3nCC_1	1	25	47	110	189	350	378
3nCC_2	1	24	52	115	185	304	329
3nCC_3	1	25	49	99	164	281	304
Total	1	34	79	182	312	594	646

2nCC_1, 2nCC_2, 2nCC_3, samples from diploid *Carassius auratus* (2nCC); 3nCC_1, 3nCC_2, 3nCC_3, samples from the triploid *Carassius auratus* (3nCC). These values represent the number of terms of each taxonomic level that have been annotated

**Fig. 1:**
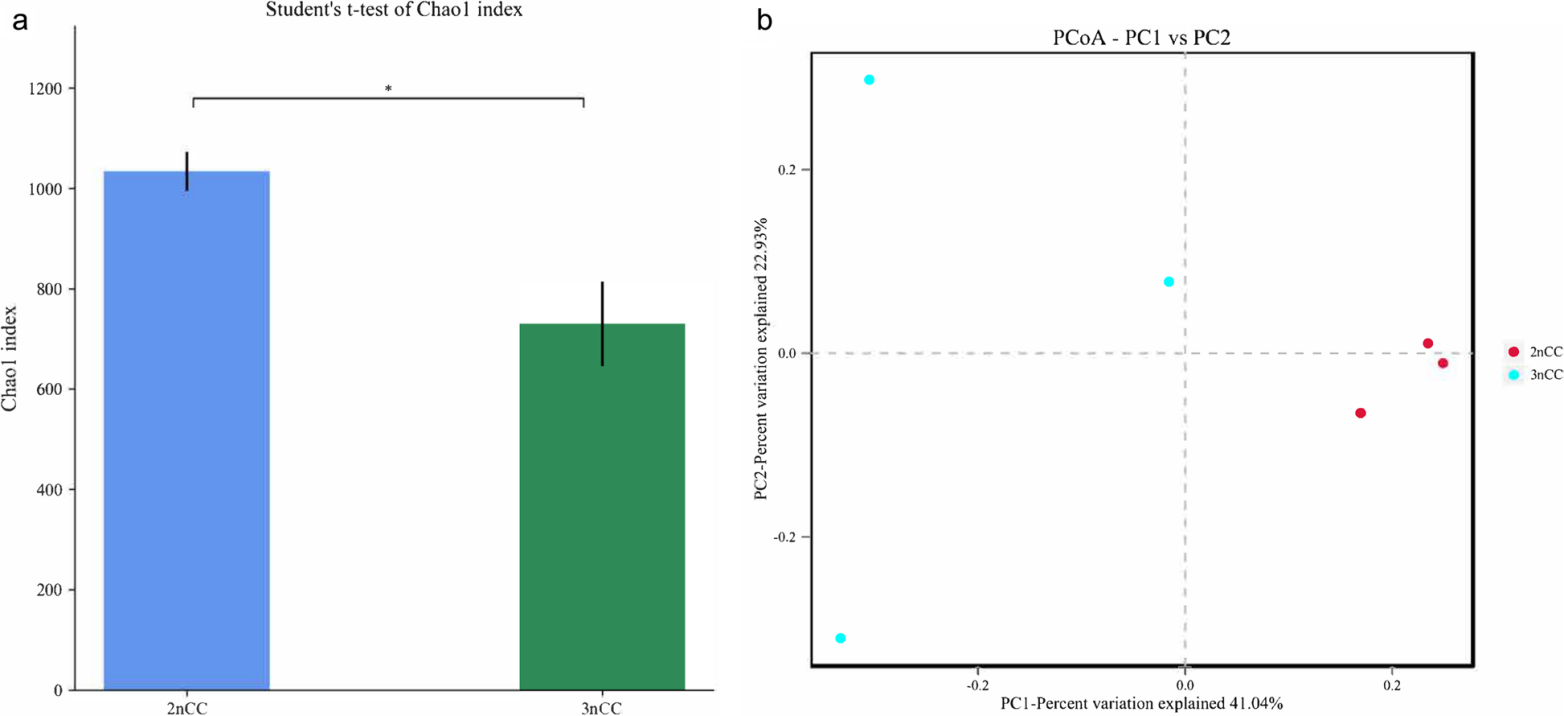
Histogram diagrams and PCoA plots illustrate responses on gut microbiota diversity and structural composition. **a** Alpha diversity (Chao1 index) of the gut microbiota in 2nCC and 3nCC. **b** PCoA ordination of unweighted UniFrac distances among crucian carp gut microbiota

For each group, we computed the Chao1 index to assess the diversity of the microbiota between diploid and triploid of *Carassius auratus* (Fig. 1a). The results imply that intestinal microbiota from the 3nCC group had significantly lower species diversity levels than those from the 2nCC group (*P* < 0.05).

PCoA distinguished the 3nCC group from the 2nCC group based on structural differences in gut microbiota from fecal samples (Fig. 1b). Triploidization changes the overall structure of the microbiota of diploid and triploid crucian carp.

### Flora differences between diploid and triploid *Carassius auratus*

**Fig. 2:**
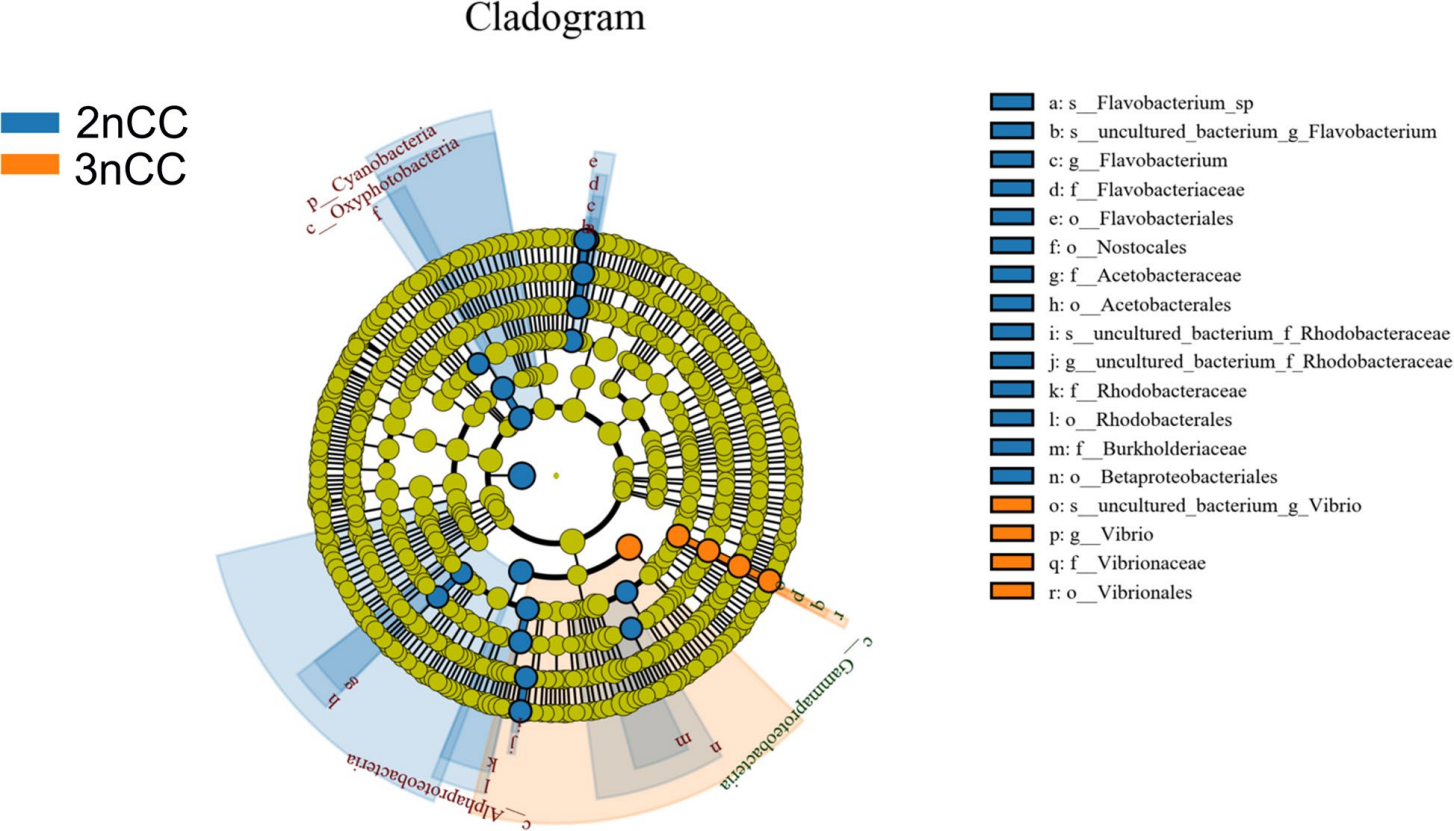
Taxonomic cladogram produced from LEfSe analysis. Blue and orange shows taxa enriched in 2nCC and 3nCC, respectively. The size of the dots is proportional to the abundance of taxon

According to the LEfSe taxon, the relative abundance of bacterial phylum, class, order, family, genus, and species differed significantly between diploid and triploid crucian carp (Fig. 2). In the 3nCC groups, the proportion of *Vibrionales* was greater than in the 2nCC groups.

Additionally, we investigated how triploidization impacted the distribution of particular flora on 2nCC and 3nCC at the order level (Table 2), family level (Table 3), genus level (Table 4), and species level (Table 5). On an order level (Table 2), *Vibrionales* greatly increased in abundance in the triploid population (*P* < 0.05) compared to 2nCC. *Rhodobacterales* were observed in 3nCC at a marginally lower abundance (*P* < 0.1) than in 2nCC. On a family level (Table 3), a significant improvement in *Vibrionaceae* relative abundance was associated with triploidization (*P* < 0.05). In 3nCC, *Rhodobacteraceae* and *Peptostreptococcaceae* (*P* < 0.1) had a marginally lower abundance than in 2nCC. On a genus level (Table 4), *Vibrio* became more prevalent in 3nCC (*P* < 0.05) than in 2nCC. We observed a marginally lower abundance in uncultured_bacterium_f_*Rhodobacteraceae* and a higher abundance in *Romboutsia* (*P* < 0.1) were observed in 3nCC vs. 2nCC. On a species level (Table 5), the relative abundance of uncultured_bacterium_g_*Vibrio* was significantly increased by triploidization (*P* < 0.05) in comparison with 2nCC. Uncultured_bacteria_ f_*Rhodobacteraceae* exhibited a marginally lower abundance in 3nCC vs 2nCC, while uncultured_bacteria_g_*Romboutsia* exhibited a markedly higher abundance (*P* < 0.1).

**Table 2:** Proportion of dominant bacteria at the order level in the 2nCC and 3nCC groups

Order Level	2nCC	3nCC	*P*-value
*Fusobacteriales*	0.213 ± 0.141	0.144 ± 0.114	0.456
*Aeromonadales*	0.056 ± 0.046	0.209 ± 0.240	0.398
*Erysipelotrichales*	0.138 ± 0.113	0.041 ± 0.002	0.285
*Bacteroidales*	0.025 ± 0.026	0.139 ± 0.132	0.305
*Clostridiales*	0.039 ± 0.046	0.074 ± 0.039	0.117
*Rhodobacterales*	0.070 ± 0.002	0.002 ± 0.001	0.094
*Vibrionales*	0.0002 ± 0.00005	0.129 ± 0.051	0.049
*Flavobacteriales*	0.059 ± 0.049	0.002 ± 0.001	0.190
*Betaproteobacteriales*	0.045 ± 0.016	0.014 ± 0.007	0.133
*Enterobacteriales*	0.008 ± 0.003	0.043 ± 0.048	0.309

Results are expressed as means ±SD

**Table 3:** Proportion of dominant bacteria at the family level in the 2nCC and 3nCC groups

Family Level	2nCC	3nCC	*P*-value
*Fusobacteriaceae*	0.213 ± 0.141	0.144 ± 0.115	0.453
*Aeromonadaceae*	0.056 ± 0.046	0.209 ± 0.240	0.398
*Erysipelotrichaceae*	0.138 ± 0.113	0.041 ± 0.002	0.285
*Barnesiellaceae*	0.002 ± 0.002	0.080 ± 0.135	0.424
*Rhodobacteraceae*	0.070 ± 0.039	0.002 ± 0.001	0.094
*Vibrionaceae*	0.0004 ± 0.0005	0.132 ± 0.034	0.022
*Flavobacteriaceae*	0.059 ± 0.049	0.001 ± 0.0001	0.183
*Enterobacteriaceae*	0.008 ± 0.003	0.043 ± 0.048	0.309
uncultured_bacterium_c_*Gam*maproteobacteria	0.011 ± 0.016	0.037 ± 0.032	0.391
*Peptostreptococcaceae*	0.014 ± 0.021	0.033 ± 0.016	0.052

Results are expressed as means ± SD

**Table 4:** Proportion of dominant bacteria at the genus level in the 2nCC and 3nCC groups

Genus Level	2nCC	3nCC	*P*-value
*Cetobacterium*	0.212 ± 0.141	0.142 ± 0.116	0.446
*Aeromonas*	0.056 ± 0.046	0.209 ± 0.240	0.398
ZOR0006	0.126 ± 0.125	0.016 ± 0.006	0.255
uncultured_bacterium_f_*Barnesiellaceae*	0.002 ± 0.002	0.080 ± 0.135	0.423
*Vibrio*	0.001 ± 0.001	0.122 ± 0.042	0.038
*Flavobacterium*	0.059 ± 0.049	0.001 ± 0.001	0.183
uncultured_bacterium_c_*Gammaproteobacteria*	0.011 ± 0.016	0.037 ± 0.032	0.391
*Romboutsia*	0.014 ± 0.021	0.030 ± 0.019	0.098
uncultured_bacterium_f_*Rhodobacteraceae*	0.037 ± 0.020	0.001 ± 0.001	0.086
uncultured_bacterium_f_*Enterobacteriaceae*	0.001 ± 0.001	0.026 ± 0.041	0.395

Results are expressed as means ± SD

**Table 5:** Proportion of dominant bacteria at the species level in the 2nCC and 3nCC groups

Species Level	2nCC	3nCC	*P*-value
uncultured_bacterium_g_*Cetobacterium*	0.212 ± 0.141	0.142 ± 0.116	0.446
uncultured_bacterium_g_*Aeromonas*	0.056 ± 0.046	0.209 ± 0.240	0.398
Firmicutes_bacterium_ZOR0006	0.125 ± 0.125	0.014 ± 0.006	0.250
uncultured_bacterium_f_*Barnesiellaceae*	0.002 ± 0.002	0.080 ± 0.135	0.423
uncultured_bacterium_g_*Vibrio*	0.001 ± 0.001	0.142 ± 0.052	0.042
uncultured_bacterium_c_*Gammaproteobacteria*	0.011 ± 0.016	0.037 ± 0.032	0.391
uncultured_bacterium_g_*Romboutsia*	0.014 ± 0.021	0.030 ± 0.019	0.098
uncultured_bacterium_f_*Rhodobacteraceae*	0.037 ± 0.020	0.001 ± 0.001	0.086
*Flavobacterium*_sp	0.030 ± 0.027	0.0002 ± 0.0003	0.192
uncultured_bacterium_g_*Flavobacterium*	0.028 ± 0.022	0.001 ± 0.001	0.173

Results are expressed as means ± SD

### Analysis of transcriptome sequences and sequence alignment

As measured by the OD ratio A260/A280 and RNA integrity numbers (RINs) of RNA of six samples, the RNA integrity numbers were 2.2 and 8.1–8.7, respectively (Additional file 1). Despite their high quality and lack of contamination, all samples underwent transcriptome sequencing. Illumina performed RNA sequencing on intestinal samples from 2nCC and 3nCC. Tables 6 and 7 display the RNA-Seq results. In six RNA-Seq libraries, the clean read count ranged between 40,639,622 and 64,300,602. The aligned clean reads were then aligned with the RCC reference genome (https://bigd.big.ac.cn/search?dbId=gwh&q=GWHAAIA00000000) using HISAT2. The mapped genome reads ranged from 33,237,039 to 57,311,126 sets, and genome map rates ranged from 80.77 to 89.22%.

**Table 6:** Overview of the RNA-Seq data collected from 2nCC and 3nCC

Sample name	Raw reads	Clean reads	Clean bases	Q20 (%)	Q30 (%)	GC content (%)
2nCC_1	41,848,936	41,848,742	6.24G	97.41	95.48	47.34
2nCC_2	40,741,902	40,741,652	6.08G	98.52	95.70	46.87
2nCC_3	42,839,398	42,839,258	6.40G	98.63	95.99	47.01
3nCC_1	64,300,916	64,300,602	9.60G	98.39	95.56	47.01
3nCC_2	40,639,814	40,639,622	6.07G	98.44	95.49	47.47
3nCC_3	41,573,124	41,572,714	6.21G	97.71	93.74	47.98

**Table 7:** Overview of clean reads mapped from 2nCC and 3nCC to the reference genome

Sample name	Total reads	Total mapped
2nCC_1	41,848,742	33,801,228(80.77%)
2nCC_2	40,741,652	33,237,039 (81.58%)
2nCC_3	42,839,258	37,652,720 (87.83%)
3nCC_1	64,300,602	57,311,126 (89.13%)
3nCC_2	40,639,622	36,258,670 (89.22%)
3nCC_3	41,572,714	36,380,282 (87.51%)

### Identification of differentially expressed transcripts (DETs) between diploid and triploid *Carassius auratus*

**Fig. 3:**
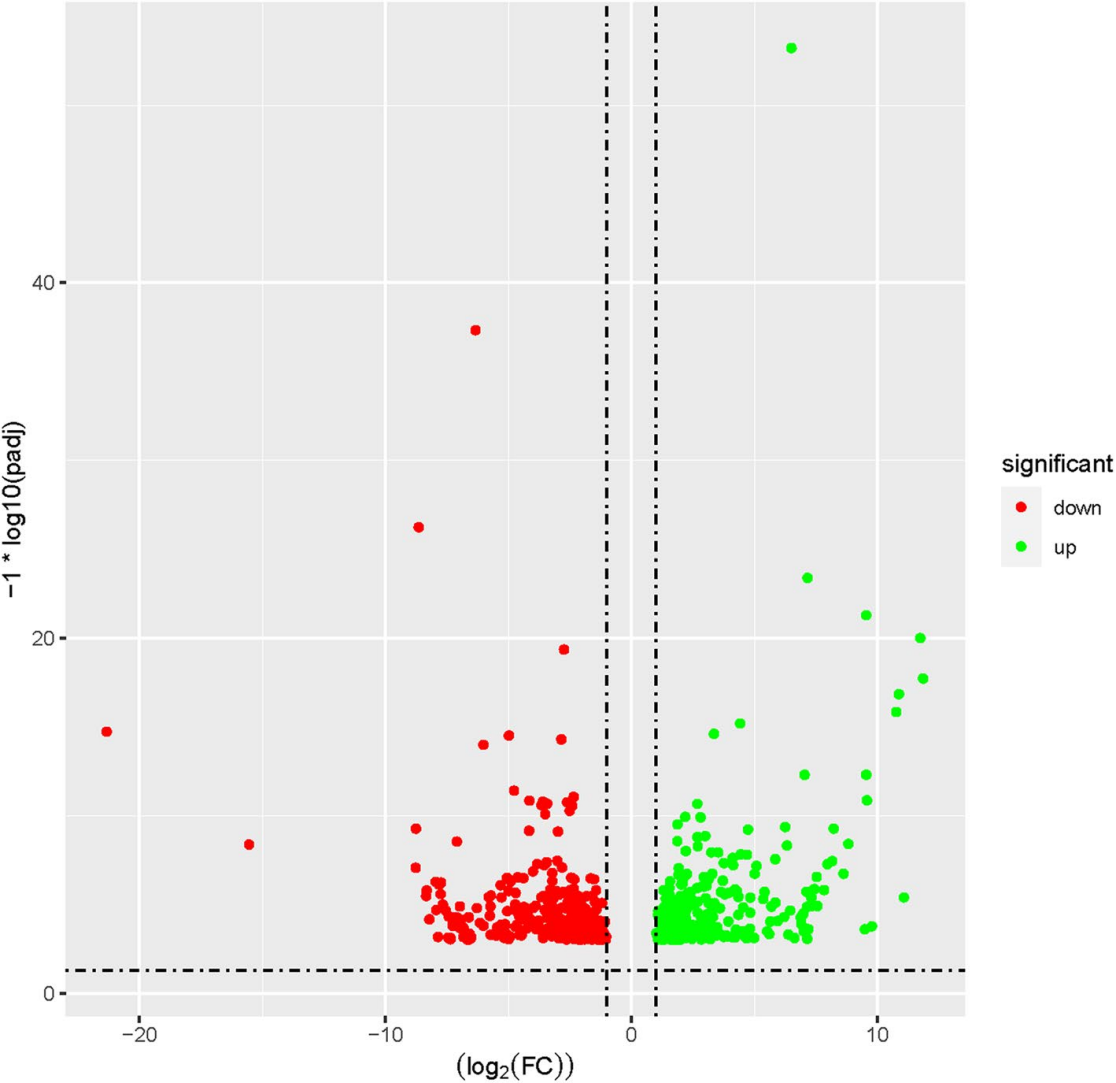
Volcano plot showing differential transcript expression between 2nCC and 3nCC

There were 293 up-regulated transcripts and 324 down-regulated transcripts observed in 3nCC and 2nCC, respectively (Fig. 3). DETs between 3nCC and 2nCC comprised osteoclast stimulatory transmembrane protein (*OCSTAMP*), leucine rich repeat LGI family member 2 (*LGI2*), upregulator of cell proliferation (*URGCP*), GIMAP family P-loop NTPase domain containing 1 (*GIMD1*), ankyrin and armadillo repeat containing (*ANKAR*), leucine rich repeat neuronal 2 (*LRN2*), G protein-coupled receptor 98 (*GPR98*), potassium channel tetramerization domain containing 7 (*KCTD7*), predicted gene 12,253 (*GM12253*), protein kinase superfamily protein (*SNRK2.4*), F-box and leucine rich repeat protein 13 (*FBXL13*), phosphorylase kinase regulatory subunit alpha 1 (*PHKA1*), leucine rich repeat containing 31 (*LRRC31*), etc.

**Fig. 4:**
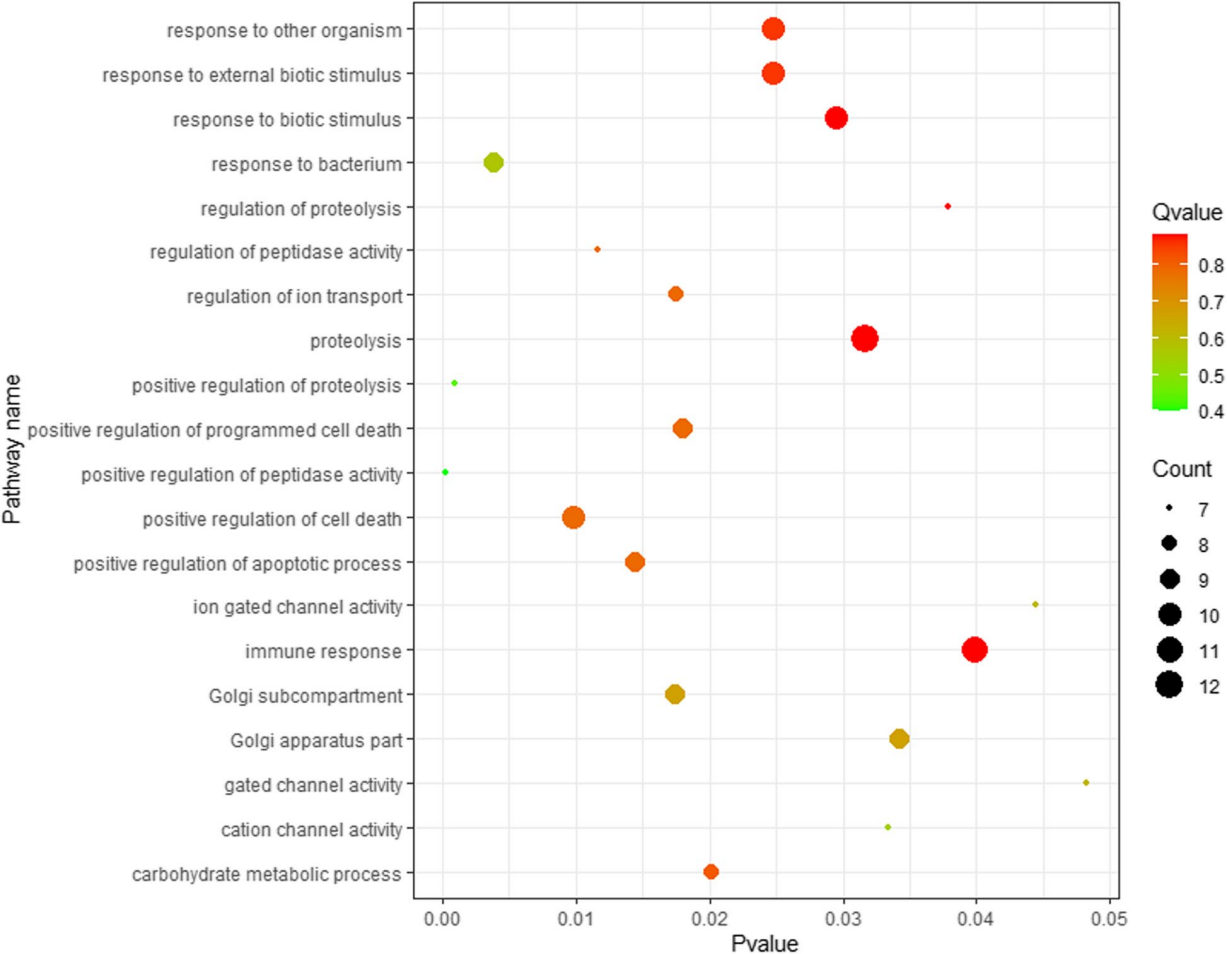
Scatter plot of enriched GO terms for DETs between 3nCC and 2nCC. Pvalue is presented on the x-axis, and the top 20 pathways are shown on the y-axis. Qvalue is the corrected Pvalue. Color scale indicates Qvalue. Circle diameters represent the number of transcripts associated

**Fig. 5:**
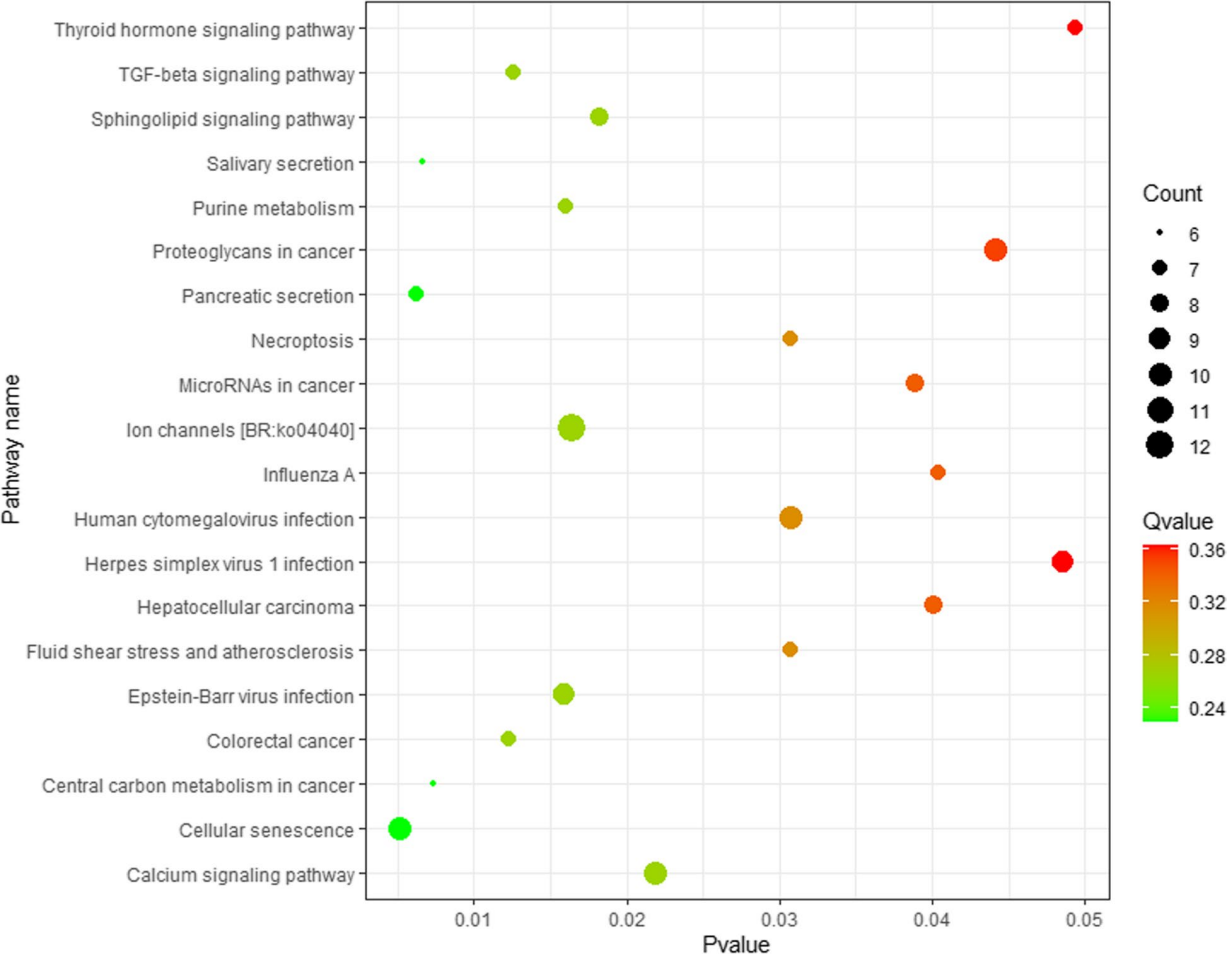
Scatter plot of enriched KEGG pathways for DETs between 3nCC and 2nCC. Pvalue is presented on the x-axis, and the top 20 pathways are shown on the y-axis. Pvalues are corrected to produce Qvalues. Color scale is used to represent Qvalues. Numbers of transcripts associated with each circle are indicated by their diameters

GO enrichment analysis uncovered 76, 8 and 16 terms, respectively, from the biological process, cellular component, and molecular function categories for the 3nCC group compared to 2nCC (Additional file 2). As illustrated in Fig. 4, the top 20 GO-terms were the most enriched, such as proteolysis, immune response, positive regulation of cell death, response to external biotic stimulus, response to other organism, response to biotic stimulus, response to a bacterium, positive regulation of apoptotic process, positive regulation of programmed cell death and Golgi subcompartment. Moreover, several pathways implicated in disease were identified, including inflammatory response, response to xenobiotic stimulus, activation of an innate immune response, inflammatory response to antigenic stimulus, and the detection of bacterium and cytokine production. DETs were enriched for 38 signaling pathways according to KEGG analysis (Additional file 3). As illustrated in Fig. 5, the top 20 KEGG pathways were enriched. Five of the most enriched pathways were ion channels, cellular senescence, calcium signaling pathway, human cytomegalovirus infection, and proteoglycans in cancer.

In conclusion, the enrichment results demonstrate that the most-enriched pathway associated with immune was immune response. The up-regulated genes included *NLRP3*, *LY9*, *PNMA1*, *MR1*, *PELI1*, and *NOTCH2*, and the down-regulated genes included *NFIL3* and *NLRC4*.

### Validation of differentially expressed transcripts (DETs) in diploid and triploid *Carassius auratus* by RT-qPCR

**Fig. 6:**
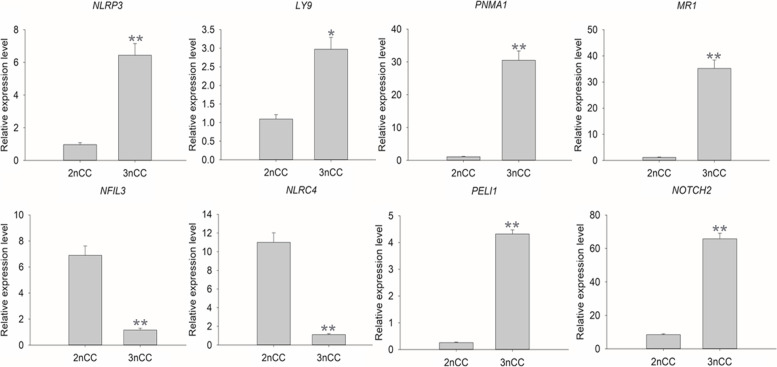
Analysis of selected DETs between 3nCC and 2nCC by qPCR. The data represent means ± SD from three independent experiments. *p < 0.05 versus control, **p < 0.01 versus control

**Fig. 7:**
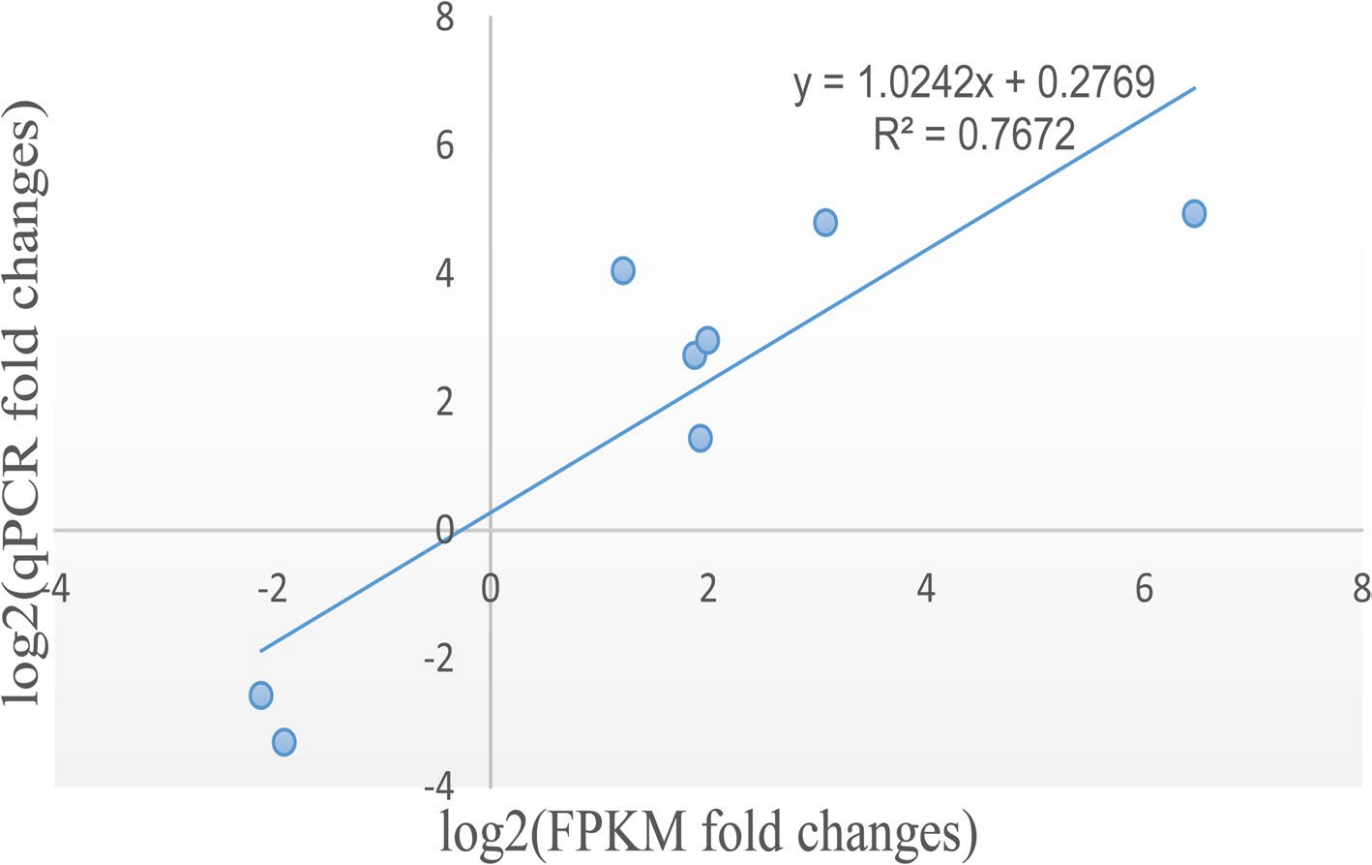
Correlation between normalized mRNA-seq results and qRT-PCR expression values. The scatterplot shows the log_2_ fold change of FPKM and qRT-PCR expression values; a trend line is shown in blue

RT-qPCR was performed to validate eight DETs based on RNA-Seq data. Six DETs that were up-regulated in the 3nCC group compared with the 2nCC group, while two DETs were down-regulated in the 3nCC group. Both RT-qPCR and RNA-Seq revealed similar expression profiles for the eight DETs (Figs. 6 and 7), indicating the reliability of the RNA-Seq results.

## Discussion

A prior study has indicated that environmental changes and variations can impact the population of diploid and triploid *Carassius auratus* [7]. In the Dongting lake water system, triploid *Carassius auratus* has been found to have a more generalized distribution and can adapt to many environments compared to diploid *Carassius auratus* [25]. This is likely because genetic and epigenetic regulation occurred in 3nCC and its adaptability to diverse environments [26, 27]. It is well-documented that the makeup of the host-microbial community is influenced by many endogenous and exogenous factors [28–31] and plays a role in regulating immune function [32]. By improving immune responses, systemic infections can be prevented [33–36]. Consequently, revealing the molecular mechanism of disease resistance from the perspective of the composition and diversity of the bacterial community in 3nCC helps us to understand its adaptability to various environments. The impact of triploidization on the microbiome and the mechanisms involved have not yet been thoroughly examined.

Triploidization raises questions about whether the immune response is influenced by the microbiota or directly by the host immune system, so we identified microbiota and gene expression in the intestines between diploid and triploid *Carassius auratus*. A shift or change in the structure and diversity of intestine microbiota was evident in this study. Moreover, 3nCC was significantly more abundant than 2nCC in the *Vibrio* genus. Numerous studies have revealed that *Vibrio* is associated with immune responses. For instance, grouper control of immunity and protection from *Vibrio anguillarum* infections can be achieved using a probiotic lactic acid bacterium *Pediococcus pentosaceus* strain 4012 [37]. An entire cDNA for a clip domain serine proteinase gene could bond to *Aeromonas hydrophila*, *Vibro anguillarum*, and *Vibro alginolyticus*, which thus reduced the pathogen-induced mortality rate [38]. The consumption of 1.0 and 2.0% *Siegesbeckia glabrescens* extract enriched diet significantly improved immune activity, improved the disease resistance of *Epinephelus bruneus* to *Vibro parahaemolyticus*, and reduced its cumulative mortality [39]. Several prior works suggested that *Vibro* is a harmful bacteria population. However, one indicates that the bacteria of the *Vibrionaceae* family (*Vibro*) are the key component of bivalve microbiota, which can cope with infectious diseases [40]. Our results suggest that triploidization can potentially alter intestine microbiota. It can be speculated that the higher abundance of *Vibro* in 3nCC may be more resistant to infections through immune response.

In this study, *NLRP3*, *LY9*, *PNMA1*, *MR1*, *PELI1,* and *NOTCH2* were the up-regulated genes, and *NFIL3* and *NLRC4* were the down-regulated genes as a result of triploidization. *NLRP3* has been demonstrated to have an important role in the inflammatory response in numerous studies. Christ et al. depicted that *NLRP3-*deficient mice were not affected by western diet-induced systemic inflammation, myeloid progenitor proliferation, and re-programming, which might have contributed to mediating the negative effects of trained immunity in inflammatory diseases [41]. Deng et al. uncovered that NLRP3 inflammasome in *Tetraodon nigroviridis* may contribute to the antibacterial immune response and produce mature TnIL-1β after activation [42]. In zebrafish, NLRP3 inflammasome has functional roles in anti-bacterial immunity [43]. In the case of *LY9*, it is independent of genetic background that *LY9*-deficient mice spontaneously developed antinuclear antibodies (ANA), anti-dsDNA, and anti-nucleosome autoantibodies, which are typical markers of systemic autoimmunity [44].

*PNMA1* falls under the family of proteins implicated in an autoimmune disorder called paraneoplastic neurological syndrome [45]. The high expression of *PNMA1* in mice is possibly a risk factor for neurodegenerative disorders [46]. Mammals possess a non-classical class I molecule, *MR1*, which serves as a sensor of microbial metabolomes and should be able to detect intracellular infection early on [47]. Many studies suggest *MR1* restricted T cells have an important role in immune contexts, ranging from cancer to autoimmunity and infection [48]. As a transcriptional regulator of immune cell differentiation, *NFIL3* is well-known [49]. According to Geiger et al., *NFIL3* plays an important cell-intrinsic role in developing gut-associated ILC3s. Additionally, *NFIL3* deficiency significantly reduces the intestinal innate immune response against acute bacterial infections such as *Citrobacter rodentium* and *Clostridium difficile* [50]. In grass carp, *NFIL3* participates in host immunity against pathogen infection and can activate various gene expressions [51]. *NLRC4* belongs to the Nod-like receptor family (NLRs), a group of cytosolic receptors that sense bacterial molecules [52]. By inhibiting the NLR pathway, Wang et al. suggested that *NLRC4* silencing alleviates lung injury and inflammation induced by septic shock [53]. Ubiquitin E3 ligase *PELI1* facilitates innate immunity and is activated by receptor signals [54]. The experimental autoimmune encephalomyelitis with mice lacking *PELI1* found that antigen presentation was enhanced, adaptive and innate immune cells were more active, and proteins involved in iron metabolism were altered [55]. *NOTCH2* is a single-pass transmembrane receptor that responds to ligands from the DSL (Delta-like family) receptors [56]. This transcription factor is expressed by various tissues and cells within the hematolymphatic compartment. It has a crucial role in the differentiation and functionality of various immunity cells [57]. Maekawa et al. demonstrated the impaired differentiation of *Notch2*-deficient T cells into cytotoxic T lymphocytes [58]. Triploidization may influence immune function by modulating the expression levels of these genes. However, it remains unclear whether changes in immune-related genes are caused either by triploidization or by an increase in Vibrio. We speculate that these immune-related genes could potentially modulate the immune response and may have a role in immune tolerance to commensal microbes, enabling 3nCC to exhibit stronger resistance to disease than 2nCC.

## Conclusions

Triploidization could modify the intestine microbiota and significantly increase the relative abundance of *Vibro*, which may, in turn, enhance disease resistance in 3nRR. We also found that eight immunity-related genes have important implications in regulating immune response and may function in immune tolerance to commensal microbes, enabling 3nCC to exhibit a stronger resistance to disease than 2nCC. The observations provide clues to decipher disease resistance in 3nCC in future work.

## Methods

### Sample preparation

In 2021, three specimens of both diploid (2nCC) and triploid (3nCC) *Carassius auratus* were sampled from the same area of Dongting Lake in Hunan Province. A flow cytometer (BD Biosciences, San Jose, California, USA) was employed to determine the ploidy type of each sample. Each fish was injected with heparinized syringes to obtain its red blood cells from its caudal vein. Staining solution (NIM-DAPI 731085) (NPE systems, Pembroke Pines, FL, USA) was added to the blood samples for 10 min. Against the DNA of red crucian carp, the total DNA content of each of the fish was compared.

After euthanizing the fish with MS-222 (100 mg/L, Western Chemical, Inc., Ferndale, Washington), the intestinal tracts were dissected with sterile scissors. Then the intestinal contents were carefully collected into sterile tubes and stored at − 80 °C for further sequencing of the 16 s rRNA. And RNA latter (Thermo Fisher Scientific, USA) was used to store the intestinal tissue shortly after collection, clearing out the contents, and storing it at − 80 °C.

### Sequencing of the 16S rRNA gene

To extract the gut microbiome DNA, MoBio’s PowerSoil DNA Isolation Kit (Carlsbad CA) was used; the quality and quantity of the resulting DNA were measured using a NanoDrop analysis method. The Illumina HiSeq 2500 library was constructed at the Biomarker Technologies Company (Beijing, China). These primers targeting the V4 and V5 regions of the 16S rRNA region were used: 338-Forward (5′-ACTCCTACGGGGGAGGCCAG) and 806-Reverse (5′-GGACTACHVGGGTWTCTAAT).

NCBI has uploaded the raw 16S rRNA sequences under BioProject ID PRJNA856111. With the aid of Trimmomatic (version 0.33), the original data were qualified, and the primer sequences were removed with Cutadapt (version 1.9.1). Following merging the paired-end reads, chimeras were removed through UCHIME (version 8.1), resulting in effective reads. Using USEARCH version 10.0, sequences with > 97% homology were classified into multiple operational taxonomic units (OTUs). Based on the SILVA reference database (version 132) and QIIME2 (version 2020.6), naïve Bayesian classifiers were employed to assign OTU sequences to SILVA representative sequences. To analyze differences in community structure between different groups, principal coordinate analysis (PCoA) was performed. Through QIIME2, the beta diversity parameters (Chao1) were calculated. At the phylum, order, class, family, genus, and species taxonomic levels, histograms were created with the R software (version 3.5.3). LEfSe (http://huttenhower.sph.harvard.edu/galaxy/) applied the nonparametric factorial Kruskal-Wallis and Wilcoxon rank-sum tests to detect significantly different species at a level of 0.05 to determine the significant difference between different groups.

### mRNA sequencing

Intestinal tissue RNA was isolated with Trizol reagent (Invitrogen) after treatment with RNase Free DNase I (Dalian Takara Co. Limited, China). A NanoDrop-2000 spectrophotometer (Implant, Westlake Village, USA) was used to measure RNA concentration and quality, and agarose (1%) gel electrophoresis was used to determine RNA integrity. cDNA synthesis and sequencing were performed using high-quality RNA from each sample. Under the manufacturer’s protocol, we constructed paired-end libraries with the TruSeqTM RNA library prep kit (Illumina, San Diego, CA, USA). Six cDNA libraries were generated by combining end-repair, 3′ end adenylation, and adapter ligation and enrichment (3 2nCC, 3 3nCC). High-throughput sequencing was performed on an Illumina sequencing platform (Illumina HiSeqTM 2500). The public repository of sequenced data at the NCBI (PRJNA857759) was considered.

Using the fastp software (version 0.20.0), adapters and low-quality reads were removed after sequencing. A quality assessment of the clean reads was performed using FastQC software (version 0.11.9), and alignments of the libraries to the RCC reference genome (https://bigd.big.ac.cn/search?dbId=gwh&q=GWHAAIA00000000) were performed with the HISAT2 tool (version 2.1.0). Fragments per kilobase per million mapped fragments (FPKMs) were used to calculate the gene expression level. DEGSeq2 R package (Version 1.28.1) was used to analyze the differentially expressed transcripts (DETs) of 3nCC versus 2nCC. DETs were genes with a fold change (FC) > 2 and false discovery rate (FDR) < 0.05. ClusterProfiler (version 3.6.0) with *p* < 0.05 was used to perform Gene ontology (GO), Kyoto Encyclopedia of Genes and Genomes (KEGG) enrichment analyses on these DETs.

### Verification of quantitative real-time PCR

A set of eight DETs (six up-regulated DETs and two down-regulated DETs) was tested using quantitative real-time (qRT) PCR to assess the reliability of 3nCC sequencing results compared with 2nCC sequencing results. Following manufacturer’s instructions, cDNA synthesis was performed using the PrimeScriptTM RT reagent kit (Takara, Dalian, China). Listed in Additional file 4 are the primer sequences for *β-actin* (the internal control gene) and these DETs. qRT-PCR reactions were conducted in a 10 μL volume using 5 μL of the SYBR Green qPCR Master Mix, 0.5 μL of 20 μM of each primer, 1 μL of cDNA (1:10 dilution), and 3 μL of nuclease-free water. As a general rule, the thermal cycle for qRT-PCR was 95 °C for 2 min, 40 cycles at 95 °C for 15 s, and annealing at 60 °C for 30 s. qRT-PCR comprised three replications per biological sample. By using the 2^−ΔΔCt^ method, we computed the relative mRNA expression levels. The data were analyzed using SPSS (v22.0) software (SPSS Inc., Chicago, IL, USA). We analyzed Students’t-tests to determine whether the results were statistically significant.

## Supplementary Information

**Additional file 1.**

**Additional file 2.**

**Additional file 3.**

**Additional file 4.**

## Data Availability

Raw RNA-Seq reads are available from the NCBI (PRJNA857759). And the obtained raw 16S rRNA gene sequences are available from the NCBI (PRJNA856111).

## Abbreviations

2nCC: Diploid *Carassius auratus*
3nCC: Triploid *Carassius auratus*
DETs: Differentially expressed transcripts
GO: Gene Ontology
KEGG: Kyoto Encyclopedia of Genes and Genomes
*NLRP3*: NLR family pyrin domain containing 3
*LY9*: Lymphocyte antigen 9
*PNMA1*: PNMA family member 1
*MR1*: Major histocompatibility complex, class I-related
*PELI1*: Pellino E3 ubiquitin protein ligase 1
*NOTCH2*: Notch receptor 2
*NFIL3*: Nuclear factor, interleukin 3 regulated
*NLRC4*: NLR family CARD domain containing 4
