# Supplementary material for: Comparative analysis of intestinal microbiota composition and transcriptome in diploid and triploid Carassius auratus
Source: BMC Microbiol. 2023 Jan 2;23:1. doi: 10.1186/s12866-022-02709-5 (PMC9806896; doi:10.1186/s12866-022-02709-5)
Supplement: Supplementary file 1 — Additional file 1. [file 12866_2022_2709_MOESM1_ESM.docx]

| Sample name | RIN | 28S/18S | OD260/280 | QC  Evaluation | Amount  (µg) |
| --- | --- | --- | --- | --- | --- |
| 2nCC_1 | 8.1 | 2.2 | 2.2 | A | 19.6 |
| 2nCC_2 | 8.5 | 2.1 | 2.2 | A | 22.3 |
| 2nCC_3 | 8.4 | 2.1 | 2.2 | A | 17.5 |
| 3nCC_1 | 8.7 | 2.2 | 2.2 | A | 18.1 |
| 3nCC_2 | 8.3 | 2.0 | 2.2 | A | 20.5 |
| 3nCC_3 | 8.2 | 2.0 | 2.2 | A | 21.6 |

Additional file 1 Quality test results of RNA
