# Supplementary material for: Comparative analysis of intestinal microbiota composition and transcriptome in diploid and triploid Carassius auratus
Source: BMC Microbiol. 2023 Jan 2;23:1. doi: 10.1186/s12866-022-02709-5 (PMC9806896; doi:10.1186/s12866-022-02709-5)
Supplement: Supplementary file 4 — Additional file 4. [file 12866_2022_2709_MOESM4_ESM.docx]

| Gene ID | Primer sequence |
| --- | --- |
| *NOTCH2*-RA-F | 5’-CATCATCGCCCATTCCAC |
| *NOTCH2*-RA-R | 5’-TGTTCAGCCTCTTCTTCTCA |
| *NFIL3*-RA-F | 5’-ATCCGACTCAAGTATGGTG |
| *NFIL3*-RA-R | 5’-CCTGTCTTTGGCGTATCT |
| *NLRC4*-RA-F | 5’-CTCTGGCTTCAGTTCTTG |
| *NLRC4*-RA-R | 5’-TCATTCCTGGGTTTCCTA |
| *MR1*-RA-F | 5’-ATGAATGGGAGAAGCACC |
| *MR1*-RA-R | 5’-GGGAATCCAGCAATCGTC |
| *PELI1*-RA-F | 5’-CCATTGATTTCGTGGTGTTG |
| *PELI1*-RA-R | 5’-GAAGCCAGCGGCGTAGAT |
| *LY9*-F | 5’-TCAGTGCTGGAGGGAGAT |
| *LY9*-R | 5’-AGAGGGAAGAGGAGCGTA |
| *NLRP3*-F | 5’-AGAGCAGGACTACGAAAG |
| *NLRP3*-R | 5’-CAGTCTCCCAGTAACAGC |
| *PNMA1*-F | 5’-AAGAAACAGCAGGGACTA |
| *PNMA1*-R | 5’-ATGATGTCTTCAGGGTTG |
| *β-actin*-F | 5’-GCCCTGCCCCATGCCATCCT |
| *β-actin*-R | 5’-AGTGCCCATCTCCTGCTCGA |

Additional file 4 Sequences of primers used in this study
